# Supplementary material for: “You have to stay in your house…because trouble can come”: The impact of education, policy, and COVID-19 on menstruation experiences in Florence, Italy
Source: PLOS Glob Public Health. 2024 Sep 20;4(9):e0003439. doi: 10.1371/journal.pgph.0003439 (PMC11414912; doi:10.1371/journal.pgph.0003439)
Supplement: S1 File — (DOCX) [file pgph.0003439.s001.docx]

**Menarche through Menopause: Understanding Menstruation Experiences in Florence, Italy**

**PI:** Andrea DeMaria, PhD, MS; Department of Public Health, College of Health and Human Sciences; Purdue University; West Lafayette, IN; USA; [ademaria@purdue.edu](mailto:ademaria@purdue.edu)

Before we begin our conversation, I want to thank you for coming today. I have a consent form for you to review before we get going, which describes the study’s purpose and details for your participation. [Hand participant two consent forms.] In brief, this study aims to understand menstruation experiences, from menarche through menopause, including menstruation management, education, and the impacts on health and well-being. Your experiences are important to us. As the interviewer, I will ask you questions related to your menstrual health, and how this relates to who you are and how you feel. As the participant, you are encouraged to share experiences you feel comfortable with, which will be kept completely anonymous. I want you to know that your opinions are very important and there are no right or wrong answers. You do not have to answer all questions, you can ask me to skip a question, and you can end the interview at any time. And if you need clarification on any questions, please do not hesitate to ask me to further explain.

I am going to give you an additional minute to further review the consent form, and please know that I am willing to answer any questions you may have. [Allow participant additional minute or two to read.] Do you understand the what the study is exploring, and what your role will be? [Allow participant to respond.] Do you have any questions for me about the study before you sign the consent form? [Allow participant to respond.] [Participant signs both. You sign both. You keep one copy, and participant keeps the other copy.] Please keep this copy of the signed consent form for your records. It contains information about the study, including the contact information for our primary investigator. Do you need anything before we get started?

Ok, I am now turning the audio recorder on, so from here forward both of our voices will be captured. Thank you, again, for agreeing to speak to me today. Just to be sure, are you okay with me audio recording today’s conversation? Please say ‘yes’ or ‘no.’

**[If they say yes, say this]:** Great, let’s begin.

**[If they say no, say this]:** Okay, thank you for your time today. Unfortunately, to be included in the study, you will need to have your interview recorded. Thank you for coming today.

What questions do you have before we get started? [wait a few moments]

Ok, great! I will now begin the audio recording. From here on, both of our voices will be captured. Just to confirm, do I have your permission to audio record today’s interview? Yes or No?

**Background:** Let’s start with some general questions about you.

(Spend approximately 3 minutes on this section.)

1. **Tell me a little bit about yourself. Where are you from/where did you grow up?**

*Probe:* Tell me about your family, or other significant people in your life.

*Probe:* Where did you go to school? What was that like?

*Probe:* What kind of work have you done in the past? Are you working right now and can you tell me about it? Or if not are you looking for work?

*Probe:* Are you from Florence? How did you/your family end up in Florence?

*Probe:* [If having lived in another town] What are the main differences between where you lived previosuly and where you live now in Florence? (i.e. small village, difference country, cultural differences)

**Menarche & Menstruation:** Next I would like to talk about menstruation, commonly referred to as ‘having a period’. (Spend approximately 10 minutes on this section.)

1. **First I would like to ask you to tell me a little bit about your first menstruation experience, also known as menarche. How old were you?**

*Probe:* Describe the memories you have associated with this experience.

*Probe:* What information did you have about menstruation? When and where did you receive this information from? Did you feel prepared?

*Probe*: [If they did not receive information] Why do you think you didn’t receive information?

*Probe:* What did you do when you first realized you were menstruating?

*Probe:* Who helped you? Who did you tell about this? [If no one] why didn’t you tell anyone?

*Probe:* What was your initial attitude toward menstruation? Has it changed over time? If so, why?

1. **How do members of your community typically talk about menstruation? With Family? Friends? Partners?**

*Probe:* Did you ever hear your mother, grandmother, or other relatives discussing menstruation when you were growing up? What was that like?

*Probe:* Did you ever hear your friends discussing mentruation? What did they say about it?

*Probe*: What did you learn from your family members, friends, or partners about menstruation?

*Probe*: How, if at all, do you feel these messages affected your menstruation experiences or attitudes?

*Probe:* How do adolescents in your community typically learn about menstruation?

*Probe:* Is menstruation something that is taboo to talk about?

1. **How is menstruation talked about in advertisements and television?**

*Probe:* How often do you see advertisements for menstruation products (tampons, pads, cups, etc.)? Where do you see these (magazine, tv, books, social media)?

*Probe:* How do you find menstruation is talked about in the media? How do you find menopause is talked about in the media?

*Probe:* What have you learned about menstruation in the media? What have you learned about menopause in the media?

*Probe:* What audiences are these media messages typically targeting?

1. **Tell me about a time you felt excited or happy about your menstrual health.**

*Probe:* What are some positive experiences you’ve had regarding menstruation? This might be bonding between family members or friends, uplifting feelings regarding the health of your body, etc.

*Probe*: How did this affect how you feel about menstruation or yourself?

*Probe*: What are some ways that you can continue having positive experiences with/attitudes toward menstruation?

*Probe*: How does menstruation help you understand aspects of your overall health?

*Probe*: Do you feel that menstrual suppression, via birth control or other forms, is healthy or unhealthy?

Why or why not?

*Probe*: Does having a regular menstrual cycle give you a sense of comfort? If so, why?

*Probe*: Do you/would you use birth control as a method of regulating your menstrual cycle? Why or why not?

*Probe*: [If they say they already do] How old were you when you began to using birth control to regulate your menstruation cycle?

1. **Have you ever purchased menstruation products from a vending machine before? This could be a small machine located in a restroom, or a larger vending machine located in a public space, like the city center. Tell me about this experience**

*Probe:* What preconceptions, if any, do you have about the menstruation products available in vending machines?

*Probe:* Have you ever had to ask a stranger for a product? What was this experience like for you?

*Probe:* Are menstruation products easy to find when you need them?

**Health & Menstruation:** Next I would like to talk about how mensturation impacts your overall health. (Spend approximately 15 minutes on this section.)

1. **What types of symptoms, positive or negative, do you typically experience with menstruation? Some examples could be increased sexual drive, changes in creativity, pain, sore breasts, irregularity, and/or heavy bleeding.**

*Probe:* How have these symptoms affected your daily life?

*Probe:* What types of things do you do to manage your symptoms? (Exercise, teas, medications, hot water)?

*Probe*: How did you learn about these symptom management strategies (e.g. medical care provider, family members, friends, online resources)

1. **What mobile apps have you used to monitor or log your menstruation experiences?**

*Probe*: What are your impressions of menstrual apps? (helpful, intrusive, etc.)

*Probe*: What feature(s) do you value most in these apps? (tracking your cycle, documenting symptoms, utilizing learning tools, etc.)

*Probe*: How, if at all, do these apps affect your attitude toward menstruation?

*Probe*: If you have not used an app, what other technologies or methods do you use? If none, why none?

1. **Have you ever talked to your healthcare provider about menstruation? Why or why not?**

*Probe*: What types of things related to menstruation have you talked about with your healthcare provider?

*Probe:* Tell me about a time you have ever felt worried or concerned about your menstrual health? Have you shared these with a doctor? Why or why not?

*Probe*: How did these conversations go? Did you feel heard, not heard? How did your healthcare provider make you feel (comfortable or uncomfortable) during these conversations, and why?

**How has the ongoing COVID pandemic impacted your menstruation experiences?**

*Probe:* Made it easier? What has helped? (bidet access, work from home in comfy clothes, easy access to own products)

*Probe:* Made it harder? How you have dealt with these challenges? (couldn’t go out to get products)

*Probe:* If you received the COVID-19 vaccine, did you notice any differences in your period?

*Probe:* Have you had to use different things for your period since the start of the pandemic? If yes, what and why was that?

**Body Image & Menstruation:** Next I would like to talk about how mensturation impacts how you feel about your body. (Spend approximately 15 minutes on this section.)

1. **I would like you to talk about your genital health and hygiene during menstruation. When I say genitals, I am referring to your vulva (or the external parts of your genitals) and your vagina (or the birth canal, where a tampon is inserted). When you think of your genitals during menstruation, what comes to mind? A clean experience? A dirty experience?**

*Probe:* Do you notice any hygiene differences between when you are menstruating and when you are not?

*Probe:* Would you say you use the bidet more, the same, or less when menstruating? Why is this?

*Probe:* Have you used products, such as a genital wash, powder, or spray during menstruation? If so, please tell me about that experience.

*Probe:* Have you ever removed your pubic hair because of menstruation (e.g., to reduce messiness, feel cleaner, feel more attractive, societal pressure, etc.)? If so, please tell me about that experience. This could be complete removal, some removal, or trimming to shorten the hair.

*Probe:* How does your sexuality change during menstruation? (more likely to have sex, decline sex, not receive oral sex)

1. **How does your La Bella Figuara change during menstruation?**

*Probe:* What does La Bella Figura mean to you?

*Probe:* How did your La Bella Figura change when you first experienced menstruation? When you experienced menopause?

*Probe:* How does La Bella Figura influence your perspective on aging?

**Italy & Menstruation Policy:** Next I would like to talk about menstruation-related policies in Italy. (Spend approximately 10 minutes on this section.)

1. **How do you feel menstruation education has changed over time in Italy? Tell me a bit about those changes.**

*Probe*: How would you say menstruation experience and attitudes differs today than in the past, if at all? Between previous and current generations? Has it ever been a “taboo” subject?

*Probe*: How have your cultural beliefs or family values impacted your perception/opinions about menstruation?

1. **What types of policies have you heard about that support menstruators?**

*Probe:* Tell me about a time you missed work or school due to menstruation. What did you do? Who did you tell?

*Probe:* Do you think there should be a policy allowing so many missed hours of work for people who experience painful or challenging menstruation symptoms? Why or why not?

*Probe:* Have you or someone you know ever had to leave a job, or change positions, due to menstruation-related reasons?

*Probe:* Who should be responsible for these policies?

*Probe:* Are there places in your community where you can get free pads, tampons, or other menstruation-related supplies?

1. **We have noticed that some hotels and public restrooms offer things like bags to dispose used products in, or genital washes next to the bidets. Are these things common to have in personal homes as well?**

*Probe:* How do you dispose of your products?

*Probe:* What products do you typically have at your home to manage menstruation and genital health and hygiene?

**Menopause:** Finally, I would like to ask about menopause. (Spend approximately 5 minutes on this section.)

1. [Ask for participants 40 and older] **Have you experienced menopause?**

Probe: [if yes] How old were you? Describe the memories you have associated with this experience.

*Probe:* What information do you have about menopause? When and where did you receive this information from? Did you feel prepared?

*Probe:* What did you do when you first realized you were going through menopause?

*Probe:* Who helped you? Who did you tell about this? [If no one] why didn’t you tell anyone?

*Probe:* What was your initial attitude toward menopause? How has this changed over time?

*Probe:* [if no] At what age to women in your family typically experience menopause? When and wehre do women typically receive information about menopause?

*Probe:* [if no]: What current thoughts and feelings do you have toward menopause?

**Concluding Remarks:** Those are all the questions I have. I appreciate your time in speaking with me, and your openness and honesty in sharing your opinions and experiences. Are there any final comments you would like to share with me before we conclude our interview?

Thank you very much for your time. To help ensure we are gathering insights from a diverse group of participants, could you please fill out a **brief demographic questionnaire** while I compile your monetary incentive documents? Because we are not revealing identities of our participants in the study, the demographic information will not be associated with you by name. Thank you!
