# Supplementary material for: “You have to stay in your house…because trouble can come”: The impact of education, policy, and COVID-19 on menstruation experiences in Florence, Italy
Source: PLOS Glob Public Health. 2024 Sep 20;4(9):e0003439. doi: 10.1371/journal.pgph.0003439 (PMC11414912; doi:10.1371/journal.pgph.0003439)
Supplement: Attachment — Submitted filename: Response to Reviewers 3.21.24.docx [file pgph.0003439.s004.docx]

Reviewer #1

This research is relevant to understand how unprecedented situations like COVID-19 pandemic would influence menstruators in terms of their work and accessibility to menstrual products. The researchers have also tried to seek details on menstruators' awareness at menarche. The methodology is quite robust and reliability of the data is ensured by the PI. A sample of 28 participants is quite adequate to give insights into diverse experience, given the geographical boundaries within which the study is undertaken. Content analysis is also undertaken meticulously.

Response>>Thank you for reviewing our paper! We appreciate your positive and constructive feedback and are grateful you can see the importance of our research in this field.

However, it is suggested that the authors address the following concerns so that whatever little gaps that appear in the manuscript can be filled in:

1. The researchers have undertaken snowball sampling to interview 28 participants for this survey. It would be good if the authors could explain in a line or two about the base sample selection (from whom snowballing was done).

Response>>This comment has been addressed. We’ve included the approximate ratios of the base sample and their recruits relative to the total number of participants. We have also reconnected this data with how these base sample participants were recruited just like the remainder of the participants- by researchers interacting with them in public places, telling them about the mission of our study, and asking them about their interest in participating.

2. The authors mention that all the participants whose interviews were audio-recorded signed an informed consent form. Was informed consent not sought from all the participants, who were interviewed? If not, give an explanation why? If yes, please mention explicitly how the consent was sought, with or without audio-recording. Informed consent is important irrespective of audio-recording or not.

Response>>This comment has been addressed. The information regarding informed consent has been clarified to include both written and verbal consent obtained and documented.

3. The phrase "education, policy and COVID-19 concerning menstruation" has been used several times in the manuscript. Do the authors mean, "awareness at menarche, menstruation leave policy and the restrictions associated with COVID-19"? It would be good to clarify this point, since this is the very objective of the study and that requires to be clearly and unambiguously stated.

Response>>This comment has been addressed. The phrase “education, policy, and COVID-19” has been clarified throughout the manuscript.

Other comments are made at relevant places in the manuscript (uploaded herewith).

Response>>All in-line comments have been addressed. Regarding the comment on line 347 questioning the positive effects that COVID-19 had on menstruation, while the results section mentions the positive impact of working from home and being more comfortable during menstruation only twice, the data found that many participants did say that they experienced the same positive impact.

Reviewer #2

As a reviewer, I congratulate the authors for presenting their research concerning a topic that requires holistic and multilevel intervention from policymakers and public health institutions worldwide.

Response>>Thank you for taking the time to review our paper! Your positive and constructive feedback is very important to us, and we are grateful for your support in increasing awareness and research on this topic.

When reviewing the paper, I came across some questions and doubts that arose during the process:

In the abstract, there needs to be more clarity regarding what the authors mean by community standards, either standards of education or standards of care and awareness.

Response>>This comment has been addressed to further describe the community standards and awareness among the community studied.

Also in the abstract, the statement: "Current menstruation education minimally supports menstruators and other European countries have started employing menstrual policies" is confusing to follow for the readers since there is no information regarding the context that the authors try to convey.

Response>>This comment has been addressed, and the sentence has been rearranged to highlight the contrast between some European countries addressing the need for a menstrual leave policy and others still failing to recognize that debilitating periods are worth educating and discussing.

In the purpose of the study presented in the abstract, there needs to be more clarity regarding the impact of COVID-19 that the authors try to investigate, whether it impacts overall well-being, menstruation patterns and symptoms, or life quality.

Response>>This comment has been addressed to reinforce our study's wholistic and multi-level aims while specifying the importance of each factor.

It is recommended that the authors explicitly state that their study is limited to a specific population within a city (Florence) rather than generalizing about "menstruators living in Italy."

Response>>This comment has been addressed and a clarification has been included to address that all participants lived within or near Florence, Italy.

Given that the study was conducted in Italy in collaboration with the Florence University of Arts, it is recommended that the authors provide a statement regarding the ethical approval (or no need for it) from the Florence University of Arts.

Response>>This comment has been addressed to note Florence University of the Arts included a letter of support in the executed research that the Purdue Institutional Review Board approved.

Did the authors consider the possible bias selection by including only English-speaking participants in a non-native English-speaking population? If so, it is recommended that the authors state how this potential bias affects their results and conclusions.

Response>>Yes, this bias was considered. We have updated the Strengths, Limitations, and Further Research section to reflect the idea that because these participants share the educational experience of learning English, they may share other important details in their background or education and, ultimately, experiences or opinions.

The authors did not mention a delimited or specified localization for the recruitment of the participants. Was there a specified consensus from the researchers regarding this topic? It is recommended that the authors comment on the selection of the participants and the recruitment site since this could affect the results.

Response>>This comment has been addressed. More information regarding where recruitment occurred has been added to the methods section.

It is recommended that the authors specify the training and requirements of the researchers in charge of data collection since the topics for the interview require specific baseline knowledge for proper guidance. Also, it is recommended that the authors specify what they mean when they mention "graduate-level research methodologies."

Response>>This comment has been addressed to include a clear description of the training level of the students and the methodologies used to gather and analyze data.

The authors mention "through being engrossed in Florentine culture." However, in the results, the authors comment about the briefness of the period the students were in the study abroad program; as well, neither in the methods section nor the results there is no specification of whether one of the researchers was originally from the selected population to state the "engrossment" within the culture.

Response>>This comment has been addressed and the methods section has been adapted to note all 28 researchers were from the United States. The Strengths, Limitations, and Further Research section has been adapted to resolve the discontinuity that the briefness of the study abroad program did not interfere with the ability of the researchers to submerge themselves in the Florentine culture and familiarize themselves with the community.

Is the protocol for the interview publicly available? Or has the questionnaire been previously validated within the bigger project mentioned by the authors?

Response>>The questionnaire was included in the research proposal and was approved by the Purdue Institutional Review Board. The interview guide is available from the corresponding author upon reasonable request, as mentioned in the title page.

Does the software used for audio recording during the interviews require a license? It is recommended that the authors make a statement regarding this concern to avoid ethical issues.

Response>>Otter is listed in the manuscript as the software used audio recording in this study. We utilized a paid Otter Pro account, and have noted this accordingly.

The authors state that the depth of knowledge and readiness regarding menstruation among the participants varied. It is recommended that the authors clarify what the outcome definition or measurement is regarding these two concepts.

Response>>This comment has been addressed. The sentence with “depth of knowledge and readiness” was deleted due to its repetitive nature. This was a different way to describe the difference in education before menarche between participants.

Given the broad age range among the participants, did the authors modify these questions according to age? Did the authors identify and search for patterns in the participants' responses in different age groups?

Response>>No changes to the interview guide were made based on participant age. The only difference in questions asked per participant were for participants who had entered menopause, as they answered an additional set of pre-approved questions relating to their experiences and opinions on menopause. The comment regarding significant differences in answers between age groups has been addressed in the discussion section, including the fact that there were no marginal differences in education, experiences, or opinions on menstruation between specific age groups.

It is recommended that the authors specify what their measured impact is. Since it is unclear whether this impact affects just the menstruation patterns or if it also includes effects on the familiar environments of the participants. Finally, the authors present "there were varying levels of impact"; however, there is no previous explanation as to what these levels might be or as to how the impact was measured since it is presented in levels.

Response>>This comment has been addressed. The comment regarding “levels” has been clarified to represent the important findings regarding COVID-19’s effects on menstruation. “Levels” did not represent how the researchers categorized the data.

The authors previously stated the need for education regarding menstruation to be imparted to “boys, men and menstruators” however, in the implications there is a statement that can be interpreted as contradictory since it is suggested that the education should be offered only for “menstruators” since the lack of the mentioned education leaves them at disadvantage.

Response>>This comment has been addressed. The discussion section has been reorganized and clarified to help clarify this topic.

It is recommended that the authors clearly stated their strengths as well as their limitations, including as to how the authors interpret that the predominant sexual orientation (mentioned as “most of the participants were heterosexual”) presented a possible limitation in interpreting the results.

Response>>This comment has been addressed to include how the predominant sexuality leads to limited sexual diversity in our data.

It is recommended that the authors unify the language to use in their paper, since the participants are described as “menstruators” while when describing the need for education regarding menstruation topics, the authors mention “boys and men”.

Response>>This comment has been addressed to unify our language.
