# Supplementary material for: “You have to stay in your house…because trouble can come”: The impact of education, policy, and COVID-19 on menstruation experiences in Florence, Italy
Source: PLOS Glob Public Health. 2024 Sep 20;4(9):e0003439. doi: 10.1371/journal.pgph.0003439 (PMC11414912; doi:10.1371/journal.pgph.0003439)
Supplement: Attachment — Submitted filename: Response to Reviewers 5.28.24.docx [file pgph.0003439.s005.docx]

Reviewer #1

Thank you for reviewing our manuscript and making sure it is suitable for your journal. We appreciate your efforts in helping us share our research with the world.

Reviewer #2

1. Reviewer #2 has raised concerns about the use of inclusive language in this study and we share the concern with the reviewer. This is a qualitative study about menstruation experiences in Italy. The words 'woman' or 'women' are only used in the quotes from the participants, however the words 'boys' and 'men' were used until they were replaced by 'non-menstruators', after the reviewer noticed this inconsistency (although not completely, see line 336).

Response>> The language, including terms like ‘boys’ and ‘men,’ has been replaced with ‘non-menstruators’ to be more inclusive and highlight that menstruation education is lacking not just among boys and men but among all who do not menstruate. A footnote has been added to address the purpose of this language.

2. We have also noticed that there is no breakdown of participants other than by sexual orientation. Please provide (1) the gender/sex breakdown of the participants, and (2) justification for the use of 'menstruators' instead of 'women' in this context.

Response>> All 28 participants identified as cisgender women on our demographic form. The other options for gender orientation that were on our demographic form were added to Table 2: Participant Demographics. Additionally, a sentence regarding the gender identity of our participants was added to the Research Participant section of the methods. A footnote explaining the use of “menstruators” instead of “women” was added to the Introduction.
